# A Simple Differential Microwave Imaging Approach for In-Line Inspection of Food Products

**DOI:** 10.3390/s23020779

**Published:** 2023-01-10

**Authors:** Noemi Zeni, Lorenzo Crocco, Marta Cavagnaro, Gennaro Bellizzi

**Affiliations:** 1 Department of Electric Engineering and Information Technologies, University of Naples Federico II, 80125 Naples, Italy; 2 Institute for the Electromagnetic Sensing of the Environment, National Research Council of Italy, 80124 Naples, Italy; 3 Department of Information Engineering, Electronics and Telecommunications, University of Rome La Sapienza, 00184 Rome, Italy

**Keywords:** microwave imaging, non-invasive diagnostics, food inspection, food security, detection

## Abstract

Microwave imaging has been recently proposed as alternative technology for in-line inspection of packaged products in the food industry, thanks to its non-invasiveness and the low-cost of the equipment. In this framework, simple and effective detection/imaging strategies, able to reveal the presence of foreign bodies that may have contaminated the product during the packaging stage, are needed to allow real-time and reliable detection, thus avoiding delays along the production line and limiting occurrence of false detections (either negative or positive). In this work, a novel detection/imaging approach meeting these requirements is presented. The approach performs the detection/imaging of the contaminant by exploiting the symmetries usually characterizing the food items. Such symmetries are broken by the presence of foreign bodies, thereby determining a differential signal that can be processed to reveal their presence. In so doing, the approach does not require the prior measurement of a reference, defect-free, item. With respect to the quite common case of homogeneous food packaged in circular plastic/glass jars, numerical analyses are provided to show the effectiveness of the proposed approach.

## 1. Introduction

In the food industry, the inspection of the integrity of food items before their commercialization has become a key step in the control of product quality. In this framework, particular attention is paid to the detection/imaging of the presence of small foreign bodies that may have contaminated food during the different production processes, particularly in the packaging stage [1].

Such an inspection can be carried out off-line, on selected samples, or directly in-line, (i.e., when the food item moves on the production line, without stopping or delaying it). The former approach is surely a less effective and more expensive solution, as the presence of even a single contaminated product requires discarding the entire batch and at the same time does not guarantee that all contaminated food is actually detected. Hence, the second solution is preferable. Currently, such task is carried out by employing metal detectors, X-ray scanners, near infrared sensors and/or optical cameras specifically designed to fit and be compliant with the production chain [2]. However, the rate of missed detections (i.e., false negatives) is still significant. As a matter of fact, metal detectors can only detect metallic objects; X-ray can fail in recognizing low-density foreign bodies, such as several types of plastics, thin pieces of glass or wood (the most common type of contaminants) and can be potentially dangerous for workers operating near the production chain, who can be accidentally exposed to radiations; infrared are limited by the short penetration depth in the sample, whereas optical cameras are viable only for transparent food and packaging.

As a result, an alternative solution allowing for the overcoming of these drawbacks is of interest. In this respect, an interesting alternative to the above diagnostic tools is represented by microwave imaging (MWI) [3,4,5,6].

MWI performs the detection/imaging relying on the difference in the dielectric permittivity characterizing the different materials making the packaged food item, in the microwave frequency range [7]. As a result, it is completely safe, making use of low intensity and non-ionizing radiations, and assures a sufficient level of penetration (hence of inspection) into the packaged food. Moreover, from a hardware point of view, MWI devices are simple and cheap [8], and can be easily integrated within an industrial production chain. Of course, it must be stressed that MWI is applicable solely to those food items packaged into non-metallic holders, which, however, represent an important percentage (if not even the majority) of the cases in the food industry.

These features motivated the recent development of a MWI system specifically meant for food inspection [9,10,11]. In its basic version, the system is essentially made of a pair of identical antennas placed on both sides of the conveyor belt of the production line, just in front of each other, at the same distance from the belt. To detect/image possible contaminants, the data collected by the antennas are processed using a differential approach where the data measured for the food item under test are subtracted to those initially acquired (and stored) for a “contaminant-free” food item used as “reference”. This differential approach assumes that all the items to be inspected are almost identical to each other, except for the possible presence of foreign inclusions. As a result, the presence of contaminants can be easily and quickly established whenever a non-null differential signal (namely, above a threshold related to the measurement noise) is detected. In addition, thanks to the fact the perturbation induced by the contaminant is electrically small and weak in terms of electric contrast, an image of the contaminant can be formed in real-time by processing the differential data by means of a linear inversion algorithm, which is a key requirement for the in-line inspection [10].

A drawback of such an approach is that the reference may be not sufficiently identical to the object under test (OUT) or, more simply, the positions at which the OUT is probed, when moving on the production line, may be not the same as the one at which the data relative to the reference object were acquired. This circumstance can increase the false positive rate, namely the wrong detection of foreign inclusions in contaminant-free products, which, while not increasing the risk of putting contaminated products on market, can represent a significant economic loss for the manufacturer, forced to discard an amount of product larger than necessary.

In the present paper, a different detection/imaging strategy is proposed, which allows one to perform the detection/imaging task without the need of a reference. The idea, preliminarily presented in [12], is still to perform a differential approach but using the OUT itself as a reference, by exploiting possible symmetries characterizing the OUT and the MWI system. More precisely, the idea is to identify possible “symmetry planes” with respect to which both the MWI system and the OUT, free of inclusions, are symmetric (from both geometrical and electromagnetic points of view). With respect to those planes, the data acquired by each of the two antennas of the MWI system are identical (except for the measurement noise) and their difference is almost zero. Then, the presence of an inclusion in the OUT destroys such a symmetry thus determining a non-null differential signal and so revealing the presence of the inclusion. A typical case of symmetric OUT, quite common in the food industry, is that of a homogeneous food, packaged in a non-metallic circular holder (e.g., a jar). With reference to such a case, in this paper two different approaches are proposed, each exploiting a different symmetry plane. The first approach, hereafter denoted as the Parallel Symmetry Plane (PSP)-based approach, exploits as symmetry plane the one parallel to (i.e., containing) the direction of movement and splitting the OUT into two identical half-parts (assuming the OUT perfectly centered between the two antennas).

The second one, hereafter denoted as the Orthogonal Symmetry Plane (OSP)-based approach, exploits as symmetry plane the one orthogonal to the direction of movement and splitting each antenna into two identical half-parts (assuming the antennas perfectly symmetric with respect to such plane).

The performances of the two approaches, either operating individually or combined together, are assessed by means of a comprehensive 3D numerical study, reproducing as faithfully as possible the real situation commonly encountered in the considered application. The robustness of the two approaches against a not perfectly centering of the OUT, with respect to the two antennas, is also investigated. 

The paper is organized as follows. In Section 2, an overview of the basic architecture of the MWI system and of the already proposed differential detection/imaging strategy based on a reference will be given first. Then, the proposed PSP and OSP based approaches will be presented. The results of the numerical analysis, assessing the effectiveness and robustness of these approaches, are provided in Section 3. Conclusions follow in Section 4. Some details on the theoretical background underlying the proposed differential MWI approaches are given in the Appendix A.

## 2. Materials and Methods

### 2.1. Overview of MWI System and of the Differential Imaging Strategy Exploiting a Reference

**Figure 1:**
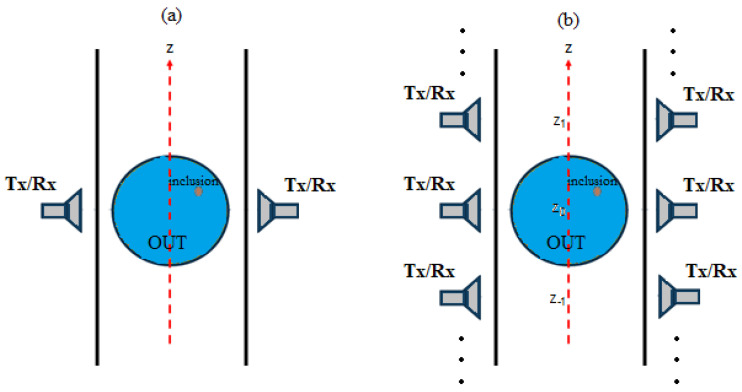
Basic scheme of the MWI system: (**a**) OUT (blue circle) moving along the conveyor belt with the two antennas at the two sides of the line; (**b**) equivalent configuration consisting of N couples of antennas, each working in bistatic mode and placed at the N measurement positions, z_n_, while the OUT stands in the middle, at the position z_0_ = 0 cm.

The basic structure of the considered MWI system is shown in Figure 1a. The system essentially consists of a couple of identical antennas placed on both sides of the conveyor belt, just in front of each other, at the same distance from the belt [9,10,11,12]. The antennas work in both transmitting/receiving mode so that one can acquire the 2 × 2 scattering matrix by means of a two-port vector network analyzer (VNA). To increase the number of independent data available, each scattering matrix is measured at M frequencies, say *f_m_* (*m* = 1, …, M). Moreover, by exploiting the movement of the OUT, each scattering matrix is measured at N different positions, say z*_n_* (*n* = −N/2, …, N/2) of the OUT along the production line, having assumed as reference position, z_0_ = 0 cm, that when the OUT is just in the middle between the two antennas. Due to the movement, this configuration is equivalent to having a synthetic array of N “virtual” pairs of antennas placed on both sides of the line, at the positions z_n_, while the OUT stands in the middle, at the position z_0_ = 0 cm (see Figure 1b).

In the equivalent configuration not all the possible pairs of scattering parameters are measured, but only those relative to the pair of antennas facing each other. As a result, the total number of independent scattering parameters acquired for each OUT is 3 × N × M (by considering the reciprocity of the transmission scattering parameters). 

In the standard approach, the collected data are subtracted to those initially measured (and stored) for an identical but contaminant-free object assumed as reference, and the difference is processed to detect/image the possible presence of undesired inclusions [9,10,11]. Specifically, denoting with Δ*S_ij_* (*f_m_*, *z_n_*), *i*,*j* = 1, 2, such a difference and with χ(*r*) the (unknown) variation of electric contrast due to the presence of the inclusion, it results (apart from an unessential multiplicative constant):

(1) $$\Delta S_{ij}\left(f_{m},z_{n}\right)=\int_{\Omega }^{}E_{bi}\left(\underline{r},f_{m},z_{n}\right)\cdot E_{bj}\left(\underline{r},f_{m},z_{n}\right)\chi \left(\underline{r}\right)d\underline{r},$$

where the domain Ω represents the OUT and **E_b_***_i_*_[*j*]_ (*i*,*j* = 1, 2) are the electric fields radiated in Ω by the *n*-th virtual couple of antennas of the synthetic array in Figure 1b, at the frequency *f_m_*.

Since the inclusion usually has a small size (compared to the wavelength) **E_b_***_i_*_[*j*]_ in (1) are well approximated by the fields radiated in the OUT in absence of the inclusion [13]. As a result, (1) describes a linear integral operator between the data and the unknown, where the kernel is given by the scalar product between the electric fields. This allows for facing the imaging as the solution of a linear inverse problem, thus enabling real time monitoring [6]. For this purpose, the truncated singular value decomposition (TSVD) scheme can be exploited [14]:

(2) $$\hat{\chi }\left(\underline{r}\right)=\sum_{l=1}^{L}\frac{1}{\sigma_{l}}<\Delta \underline{s}\cdot {\underline{u}}_{l}>v_{l}\left(\underline{r}\right),$$

where $\hat{\chi }$ is the estimated unknown contrast, Δs the differential data in (1) organized as a column vector, the triple {*σ_l_*, *u_l_*, *v_l_*} the singular value decomposition of the integral kernel in (1), and L the truncation index representing the regularization parameter of the linear and ill-posed inverse problem in (1) [14]. L is chosen as a trade-off between accuracy and stability of the reconstruction [14].

### 2.2. Differential Detection/Imaging Strategy Exploiting Symmetries

#### 2.2.1. PSP-Based Approach

**Figure 2:**
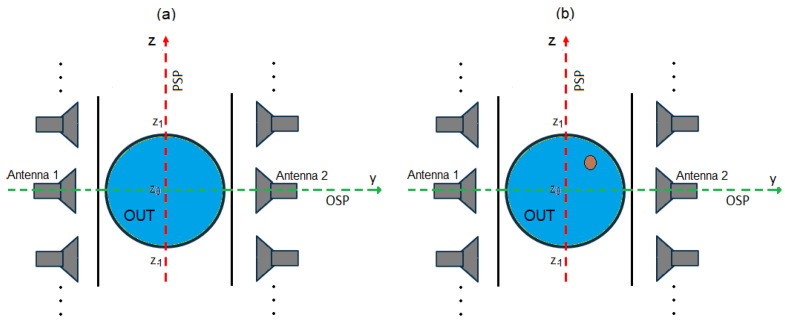
Symmetries exploited by the proposed detection/imaging approaches: (**a**) the OUT is free of inclusions; (**b**): the OUT presents an inclusion so that the symmetry is lost.

The idea underlying the PSP-based approach is depicted in Figure 2. The considered case is quite common in the food industry and is based on an OUT consisting of homogeneous food (such as yogurt, oil, mayonnaise, milk, etc.) contained in a plastic/glass holder of circular shape, located at the same distance from each of the two antennas of the N virtual couples of antennas of the synthetic array.

As it can be seen from Figure 2a, in absence of inclusions in the OUT, the overall system (OUT plus synthetic array) is perfectly symmetric with respect to the PSP (i.e., the xz-plane in figure). Accordingly, the scattering parameters, and in particular the reflection coefficients S_11_(z*_n_*) and S_22_(z*_n_*), measured at the ports of each couple of antennas, are identical (apart from the measurement noise). On the other hand, when an inclusion, not located on PSP, is present in the OUT (Figure 2b), the symmetry is broken and diversity in the measured S_11_(z*_n_*) and S_22_(z*_n_*) takes place. Therefore, by performing the difference between S_11_(z*_n_*) and S_22_(z*_n_*), one can immediately establish the presence of inclusions by simply comparing such difference with a prescribed threshold related to the noise and other systematic errors affecting the measurement (such as a not perfect symmetry in the antenna behavior). In addition, such a differential data can still be exploited to form an image of the inclusion by exploiting (2). In fact, the relationship between the unknown χ and the differential data is still given by (1) with ΔS*_ii_* = S*_ii_*(z*_n_*) − S*_jj_*(z*_n_*) (*i* = 1, 2 and *j* = 2, 1). Accordingly, by exploiting the symmetry of the OUT, it is no longer necessary to employ a “reference” object to perform the differential imaging as the reference becomes the OUT itself.

It is worth noting that the proposed strategy fails when the inclusion lies on (or is very near to) the PSP in Figure 2. However, this drawback can be mitigated by exploiting other symmetries of the OUT and MWI system (if any, as happens for case at hand), as it will be shown in next subsection.

Another inconvenience occurs when the OUT is not perfectly in the middle but is slightly displaced toward one of the two antennas. In this case, the symmetry is lost even in absence of inclusion and the approach no longer works. In this case, as shown in Section 3.3, the effects of possible offsets of the OUT from the middle can be compensated by properly “calibrating” the measured S_11_ and S_22_, provided the displacement is known.

#### 2.2.2. OSP-Based Approach

From Figure 2a one can note that, for a contaminant-free OUT, the overall system is symmetric also with respect to the OSP (i.e., the xy-plane in figure) provided that the employed antennas are symmetric with respect to such plane (as, for instance, it happens for horn antennas). As a result, the scattering parameters measured by the pair of antennas located at the position z*_n_* and those measured by the pair of antennas located at the mirror position, z*_−n_* (with respect to the OSP) are almost identical. Again, when an inclusion, not located on the OSP, is present in the OUT (see Figure 2b), the symmetry is lost and a diversity in the scattering parameters measured at z*_n_* and z*_−n_*, arises. Therefore, by performing the difference between S*_ij_*(z*_n_*) and S*_ij_*(z*_−n_*), *i*,*j* = 1, 2, one can immediately establish the presence of inclusions by simply comparing such difference with a threshold dictated by the measurement noise. In addition, such differential data can be exploited to form an image of the inclusion by exploiting (2), as the relationship between the unknown χ and the differential data is still given by (1) with ΔS*_ij_* = S*_ij_*(z*_n_*) − S*_ij_*(z*_−n_*).

Similar to the PSP-based approach, this approach fails when the inclusion lies on (or is very near to) the OSP in Figure 2. However, this drawback can be mitigated by combining both the approaches. By doing so, the above drawback is limited solely to the points intersecting both PSP and OSP.

In addition, unlike the PSP-based approach, the detection capability of the OSP-based approach is not impaired in the case the OUT is not perfectly centered but is slightly displaced toward one of the two antennas. This happens because the displacement of the OUT from the center is along the OSP itself, and this does not affect the symmetry. However, the knowledge of the entity of the displacement is still required to perform the imaging as this information is needed to evaluate the right Green’s functions to be employed in the inversion algorithm (see Section 2.4).

### 2.3. Numerical Set-Up

To assess the effectiveness of the PSP and OSP based approaches, we carried out a realistic 3D numerical analysis by reproducing as faithfully as possible the real situation commonly encountered in the considered application. The software employed for the simulation is the CST Microwave Studio version 2020 (provided by Dassault Systems).

**Figure 3:**
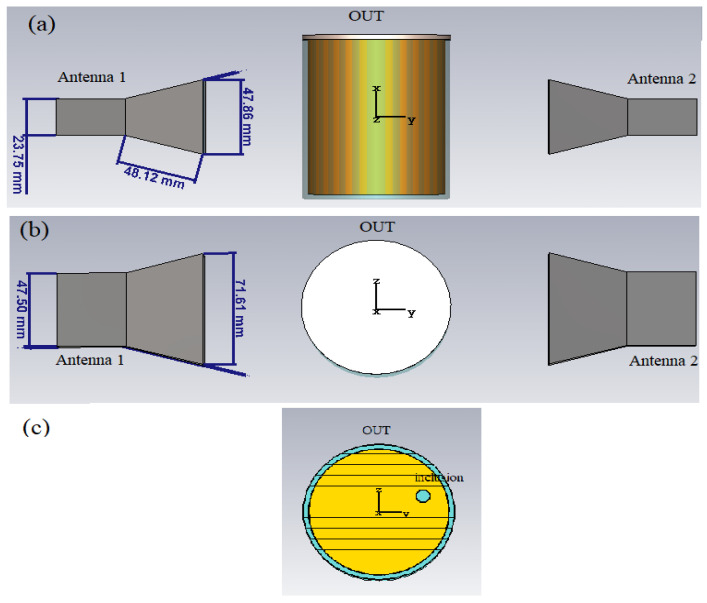
Numerical set up adopted in the simulations: (**a**) front view; (**b**) top view; (**c**) a cut of the of the OUT.

**Figure 4:**
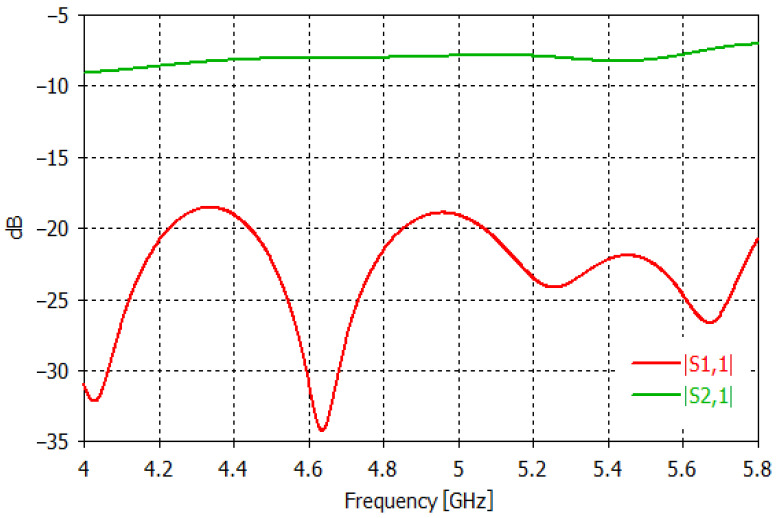
|S_11_|and |S_21_| (in dB) vs. frequency simulated at the antenna ports, in absence of the OUT.

Figure 3 shows the set up adopted in the simulations. As it can be seen, the radiating system consists of two identical WR-187 horn antennas facing each other (along the *y*-axis of the fixed reference system), at a distance of 20 cm (between the two apertures). With respect to the xz-plane (representing the PSP), each antenna is exactly in the mirror position to the other. This assures a perfect symmetry of the radiating system, hence of the radiated electric field, **E**, with respect to the xz-plane, in agreement with the requirements of the PSP-based approach. The choice of horn antennas also assures a high symmetry of the radiated electric field with respect to the xy-plane in Figure 3 (representing the OSP), thanks to the perfect geometrical symmetry of such type of antennas with respect to such plane, which is essential to exploit the OSP-based approach. In addition, horn antennas are easy to make (and to simulate) and have a relatively large bandwidth (4.0 ÷ 5.8 GHz for the adopted WR 187 horn antennas), which make them suitable for acquiring multi-frequency data. It is worth noting that in our study the size of the horn (see Figure 3) is smaller than the standard one that is commercially available. This choice allows for having both a more compact antenna and a less directive radiated field so to cover a larger region of space. This last circumstance is important as it enables collecting data with a higher spatial diversity, since the OUT is probed at more different positions during its movement on the conveyor. In addition, the size of the horn is such to assure a good return loss (below −18 dB over the entire band of interest) and a good coupling between the two antennas (above −9 dB over the entire band of interest), as one can see in Figure 4 showing, respectively, the magnitude of the simulated reflection and transmission coefficients at the antenna ports.

In any case, it is worth noting that the choice of the specific radiating elements is not essential since the only requirement is the symmetry between the antennas and of the antenna itself with respect to the E-plane. As such, other type of antennas (in terms of radiating features, bandwidth, return loss and so on), specifically designed/optimized for the application at hand, can be employed.

The OUT consists of a holder of circular shape having inner radius r = 40 mm, height h= 100 mm and thickness t = 3 mm, simulating a glass jar (see Figure 3). The relative permittivity is that of glass, ε_r_ = 6 over the whole band of interest. The jar is meant to be filled with a homogeneous medium having complex relative permittivity ε_r_ = 3 − j0.42. The latter value is that measured in [10] for a commercially available hazelnut–cocoa cream, through an open-ended coaxial probe [15], and it is the value typically expected for food with high fat content (such as oil, mayonnaise, chocolate cream, etc.). The jar is sealed on the top by a plastic cap, having relative permittivity ε_r_ = 2.1. Finally, to simulate the presence of a solid contaminant, we have inserted in the food a small glass inclusion (ε_r_ = 6) of 8 mm in size, at the position (0, 25, 10) mm (see Figure 3c).

The OUT is put just in the middle between the two antennas, at a distance of 57 mm from each of them. The movement of the OUT along the production line was simulated by moving it each time at the desired position and running the simulation. The presence of the conveyor belt was also simulated through a metallic plane put just under the OUT.

The frequencies and the positions at which the OUT was probed are: *f_m_* = 4.0 ÷ 5.8 GHz, with a step Δ*f* = 0.18 GHz (*m* = 1,…, 11, for a total of M = 11 frequency samples), and z*_n_* = −6 ÷ 6 cm, with a step Δz = 1 cm (*n* = −6,…,6, for a total of N = 13 positions. Such positions refer to the OUT center), having assumed as negative the positions of the OUT moving toward the antenna system, as positive the positions of the OUT moving away from the antenna system, and as z_0_ = 0 cm the position of the OUT when it is just in front of the two antennas (i.e., as shown in Figure 3b).

In conclusion, let us note that, except for the frequency range (herein set lower in order to increase the simulation speed up) and the antennas (chosen according to the frequency range), the above setting is the same as in [10]. In [10], the positions z*_n_* was set by assuming an average longitudinal spacing between two adjacent OUTs of approximately 8 cm (i.e., of approximately 16 cm if measured at the OUTs centers), corresponding to one jar. This assures that when the antenna system starts probing the n-th OUT, at the position z_−N/2_ = −6 cm (such position refers to the center of the OUT), the object preceding the OUT on the line is entirely beyond the position z_N/2_ = +6 cm. If, as in this case, the antenna “directivity” and spacing is such to have a negligible radiated field outside the range = −6 ÷ 6 cm, the contribution of the adjacent objects to the scattered field, as well as their coupling with the OUT, can be neglected. This enables one to treat each OUT as independent, i.e., an isolated, object on the line.

### 2.4. Numerical Green’s Functions and Inversion Procedure

Since the inclusion is expected to be a weak scatterer, its reconstruction from differential data reduces to the solution of a linear inverse problem where the Green’s function is that evaluated in presence of the OUT free of inclusions (see also Appendix A). Let us note that for the considered set-up the radiated electric field is mostly linearly polarized and directed along the axis of the OUT, namely the *x*-axis, so that hereafter only such component, say E_bx_, of the radiated electric field will be considered.

Since the OUT is probed at M frequencies and N positions, the total number of simulated E_bx_ for each antenna is P = M × N, each of which is stored in a matrix of Q (=50 × 40 × 40) elements representing $\sqrt{f_{m}}$E_bx_(x, y, z) evaluated on a grid of cubic cells (2 × 2 × 2 mm in size) into which the OUT is discretized (i.e., x = −h/2, … h/2, y = −r, …, r and z = −r, …, r, with a step size of 2 mm along each axis). As the differential data are organized as column vectors of P elements, each of the above matrix was recast as a row vector of Q elements and stacked in a P × Q matrix representing (apart from an unessential multiplicative factor) the numerical Green’s function employed in the inversion algorithm. Since the system consists of two antennas, we computed two Green’s functions, say $\underset{=}{G}$_1_ and $\underset{=}{G}$_2_, where, by virtue of the assumed symmetry, $\underset{=}{G}$_2_ can be computed by “mirroring” $\underset{=}{G}$_1_ with respect to the *y*-axis, or vice versa.

Now, regarding the PSP-based approach, denoting with s_R_ the Px1 (i.e., the column) vector of the differences between the measured *S_ii_*(*f_m_*, z*_n_*) and S*_jj_*(*f_m_*, z*_n_*) (*i* = 1, 2 and *j* = 2, 1) and with $\underset{=}{K}$*_i_* the Hadamard-Schur product [16] of $\underset{=}{G}$*_i_* by itself (*i* = 1, 2), the numerical counterpart of (1) is:

(3) $$\left[\begin{array}{c} +{\underline{s}}_{R}\\ -{\underline{s}}_{R} \end{array}\right]=\left[\begin{array}{c} {\underset{=}{K}}_{1}\\ {\underset{=}{K}}_{2} \end{array}\right]\cdot \underline{\chi }$$

being *χ* the Qx1 vector of the searched unknown contrast in the OUT (representing the inclusion).

Concerning the OSP-based approach, denoted with s*_ii_* the Px1 vector of the differences between *S_ii_*(*f_m_*, z*_n_*) and S*_ii_*(*f_m_*, z_−*n*_) (*i* = 1, 2), with s_21_ the Px1 vector of the differences between S_21_(*f_m_*, z*_n_*) and S_21_(*f_m_*, z_−*n*_) and with $\underset{=}{K}$*_ij_* the Hadamard-Schur product of $\underset{=}{G}$*_i_* by $\underset{=}{G}$*_j_* (*i*,*j* = 1, 2), the numerical counterpart of (1) is:

(4) $$\left[\begin{array}{c} {\underline{s}}_{11}\\ {\underline{s}}_{22}\\ {\underline{s}}_{21} \end{array}\right]=\left[\begin{array}{c} {\underset{=}{K}}_{11}\\ {\underset{=}{K}}_{22}\\ {\underset{=}{K}}_{21} \end{array}\right]\cdot \underline{\chi }$$

For both the PSP and OSP based approaches, *χ* is solution of a (ill-conditioned) linear system of equations. An estimate of *χ* can be then achieved by a TSVD inversion algorithm, according to the scheme (2), by choosing a proper truncation threshold for the singular values of the matrixes appearing in (3) and (4). In our reconstructions, we have set a value for the truncation threshold of −40 dB below the largest (i.e., the first) singular value.

## 3. Results and Discussion

### 3.1. Results Relative to the PSP-Based Approach

**Figure 5:**
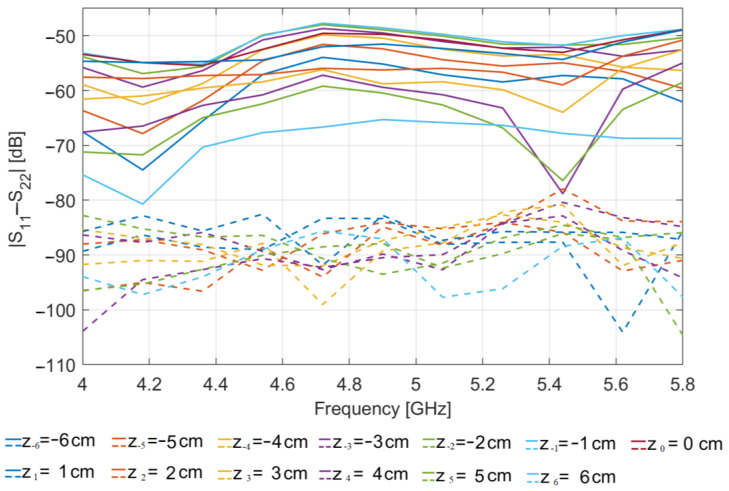
Simulated |S_11_(z*_n_*)–S_22_(z*_n_*)| (in dB) vs. frequency: OUT with an 8 mm glass inclusion (solid lines); OUT free of inclusions (dashed lines).

Figure 5 shows the magnitude (in dB) vs. frequency of the difference between S_11_(z*_n_*) and S_22_(z*_n_*) simulated at the two antenna ports, for each of the positions z*_n_* at which the OUT is probed, when the glass inclusion is present in the OUT (solid lines). For comparison, Figure 5 also reports the same plots obtained when no inclusion is present (dashed lines). 

As it can be seen, the values of |S_11_−S_22_| obtained in presence of the inclusion are appreciably larger than those in absence of it, clearly indicating that the presence of the glass inclusion breaks the symmetry of the OUT (and of the MWI system) with respect to the xz-plane, thereby determining an appreciable difference in the reflection coefficients measured at the two antenna ports, not detected in absence of inclusion. From the figure, one can also note that the level of such difference variates with the position at which the OUT is probed: it is higher when the inclusion is nearer to the antennas and lower when the inclusion is farer. This is an expected result due to the directivity of the employed antennas and confirms that for the application at hand a more convenient choice is that of employing poorly directive antennas.

The detected differential data can be then processed to establish the presence of the inclusion (detection) and possibly to image it by means of the linear inversion algorithm described in Section 2.4. To simulate the effect of the measurement noise, we added to the data a white Gaussian noise with a level of −65 dB (which is consistent with the noise level characterizing commercially available VNAs).

**Figure 6:**
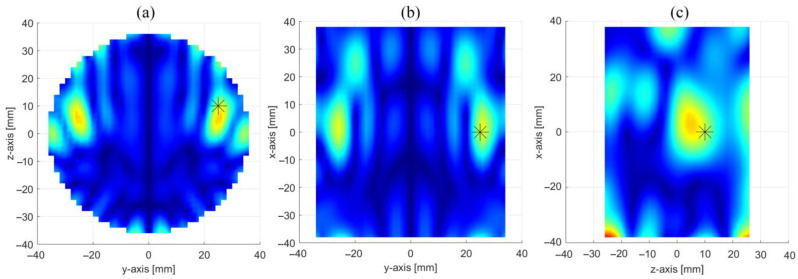
Reconstruction of the inclusion through the PSP-based approach: images in three orthogonal cut planes crossing the center of the inclusion (stars give the position of the inclusion), i.e., (**a**) x = 0 cm cut plane, (**b**) z = 10 cm cut plane and (**c**) y = 25 cm cut plane.

Figure 6 shows the achieved reconstruction in three orthogonal cut planes crossing the center of the inclusion. From panel (a) one can clearly note the presence of two main spots: a first one, at the same position of the inclusion, representing the inclusion itself, and a second one, perfectly specular to the former with respect to the xz-plane, which is the “ghost” arising from the symmetrization introduced by the PSP-based approach (see Section 2.2.1 and Appendix A). As already stressed in Section 2.2.1 and Section 2.2.2, this ambiguity can be settled by exploiting other symmetries of the OUT and radiating system, such as the one with respect to the xy-plane, exploited by the OSP-based approach. This will be shown in Section 3.3.

Apart from the presence of the ghost, a satisfactory reconstruction is obtained, with the inclusion better localized in the yz (i.e., the horizontal) cut plane than the other two (i.e., the vertical ones). This is an expected result and is due to the different positions, z_n_, at which the OUT is probed when moving along the production line (represented in the set-up by the *z*-axis). Indeed, as pointed out in Section 2.1, while we have only one couple of antennas, the movement along the *z*-axis allows for creating a synthetic array of N virtual couples of antennas along the *z*-axis, which increases the spatial diversity, hence the resolution, along such axis.

The above results refer to a glass inclusion of 8 mm in size. The smallest size of the inclusion that can be effectively revealed depends on several factors. One of them is the measurement noise, here assumed with a level of 65 dB below the level of the signal feeding each antenna. Since the level of the signal scattered by the inclusion, i.e., the collected differential data, decreases as the third power of the size, this implies that an inclusion smaller than the one considered in this study can be revealed by lowering the noise level. For instance, it is expected that an inclusion of 4 mm in size (half of the size considered above) can be detected if the noise level does not exceed −83 dB, which is a very low value, but not prohibitive for the measurement instruments commercially available. However, it must be noted that other factors influence the detection capability of the system and so the minimum size of the detectable inclusion. The material of the inclusion (glass, plastic, metal, wood and so forth), namely its electric contrast with respect to the food, is one of them. The higher the contrast, the smaller the size of the inclusion that can be detected. A further factor is the antenna return loss, that in this study is below −18 dB over the entire analyzed frequency band. A lower return loss ensures a higher radiated power, hence a higher level of the signal scattered by the inclusion, and so a higher signal to noise ratio for a given inclusion size. In any case, we retain that an inclusion of 4 mm in size can be confidently revealed as, on the other hand, it is shown by the experimental results reported in [10,17].

In conclusion, it is worth noting that the implementation of such an approach, in addition to requiring two almost identical antennas, requires a perfect alignment of the two in order to ensure a perfect symmetry of the radiating system with respect to the xz-plane.

### 3.2. Results-Relative to the OSP-Based Approach

**Figure 7:**
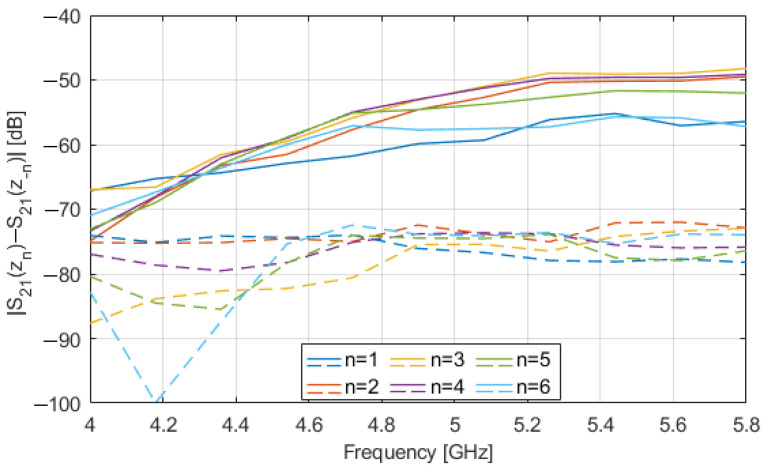
Simulated |S_21_(z*_n_*) − S_21_(z_−*n*_)| (in dB) vs. frequency: OUT with an 8 mm glass inclusion (solid lines); OUT free of inclusions (dashed lines).

Figure 7 shows the magnitude (in dB) vs. frequency of S_21_(z*_n_*) − S_21_(z_−*n*_), for z*_n_* = 1 ÷ 6 cm, with (solid lines) and without (dashed lines) the glass inclusion in the OUT. The values of the difference obtained in presence of the inclusion are appreciably larger than those without it. Again, this result is due to the presence of the glass inclusion, which, in this case, destroys the symmetry of the OUT (and of the MWI system) with respect to the xy-plane, thus leading to a difference in the transmission parameters measured at the two mirror positions, z*_n_* and z_−*n*_, assumed by the OUT in moving along the *z*-axis. The same trend is observed for |S_11_(z*_n_*) − S_11_(z_−*n*_)| and |S_22_(z*_n_*) − S_22_(z_−*n*_)| but not reported for the sake of brevity.

These differential data can be then exploited to detect the presence of the inclusion and possibly to image it by means of the linear inversion algorithm described in Section 2.4. Again, the effect of the measurement noise was considered by adding to the data a white Gaussian noise with a level of −65 dB.

**Figure 8:**
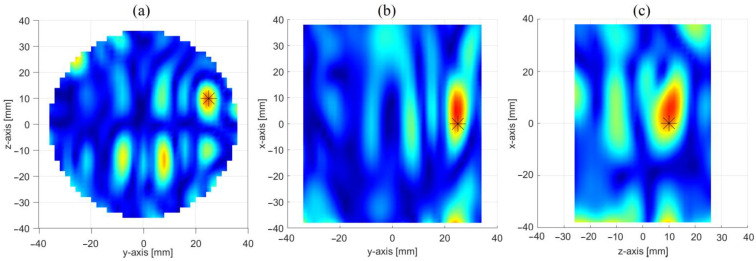
Reconstruction of the inclusion through the OSP-based approach: images in three orthogonal cut planes crossing the center of the inclusion (stars give the position of the inclusion) i.e., (**a**) x = 0 cm cut plane, (**b**) z = 10 cm cut plane and (**c**) y = 25 cm cut plane.

Figure 8 shows the achieved reconstruction in three orthogonal cut planes crossing the center of the inclusion. Similar to the PSP-based approach, from panel (a) one can note the presence of a two sets of spots: a first one, on the top part of the image, i.e., the most intense one, which represents the inclusion (the other spots are artefacts due to the inversion procedure); and a second one, specular to the former, with respect to the xy-plane, which arises from the symmetrization introduced by the OSP-based approach. As it will be shown in the next section, this ambiguity can be settled by properly combining the maps in Figure 6 and Figure 8.

In this case as well, leaving aside the ghost, a good reconstruction is obtained, with the inclusion better localized in the yz (i.e., the horizontal) cut plane than the other two (i.e., the vertical ones). Of course, this approach fails when the inclusion lies on the xy-plane (i.e., on OSP), as in this case the symmetry is preserved even in presence of it. However, this drawback can be alleviated by exploiting together the PSP and OSP-based approaches.

In conclusion, it must be stressed that the implementation of such an approach, in addition to requiring almost identical antennas and a perfect aligning of them, requires a perfect synchronization between the acquisition system and the movement of the OUT in order to acquire the scattering parameters exactly at the mirror positions, z_−*n*_ and z*_n_*, along the *z*-axis. In any case, the knowledge of the displacement is still required to perform the imaging as this information is needed to evaluate the right Green’s functions to be employed in the inversion algorithm (see Section 2.4).

### 3.3. Results Combining the PSP and OSP Based Approaches and Robustness Analysis

As already pointed out, while the PSP and the OSP based approaches can work individually, their combination can be fruitfully exploited to prevent the onset of “ghosts”, hence the ambiguity, due to symmetrization, as well as to reduce the failure of the approaches when the inclusion lies on the respective symmetry planes.

**Figure 9:**
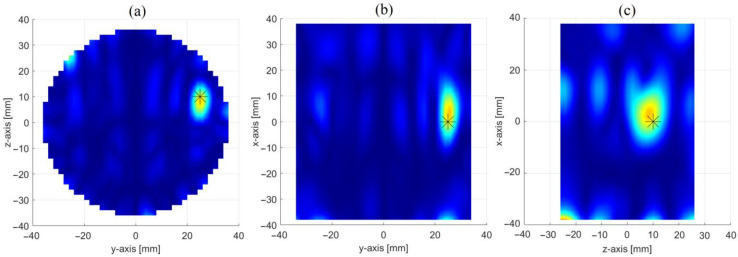
Reconstruction of the inclusion by combining the results of the PSP and the OSP based approaches: images in three orthogonal cut planes crossing the center of the inclusion (stars give the position of the inclusion) i.e., (**a**) x = 0 cm cut plane, (**b**) z = 10 cm cut plane and (**c**) y = 25 cm cut plane.

Figure 9 shows the reconstruction obtained by combining the maps in Figure 6 and Figure 8. Specifically, the resulting map is the point-by-point product of the two maps obtained separately through the PSP and OSP based approaches. As foreseen, the ghosts are strongly attenuated, thus making the imaging of the inclusion no longer ambiguous.

Moreover, the failure region is restricted to the only central region of the OUT, namely to the points along the *x*-axis (intersection of PSP and OSP).

Finally, the robustness of both the PSP and OSP based approaches was assessed when the OUT was not perfectly in the middle between the two antennas, but it was slightly displaced toward one of them. Specifically, a displacement along the y axis of *d* = 5 mm from the middle was considered.

**Figure 10:**
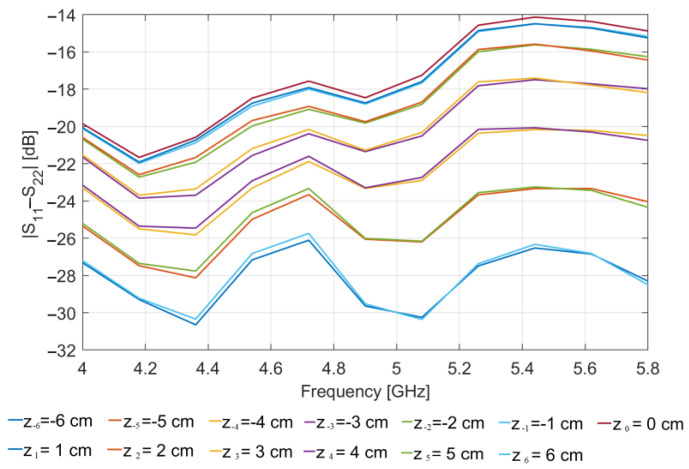
|S_11_ − S_22_| (in dB) vs. frequency simulated when the OUT is displaced of +5 mm along the *y*-axis.

**Figure 11:**
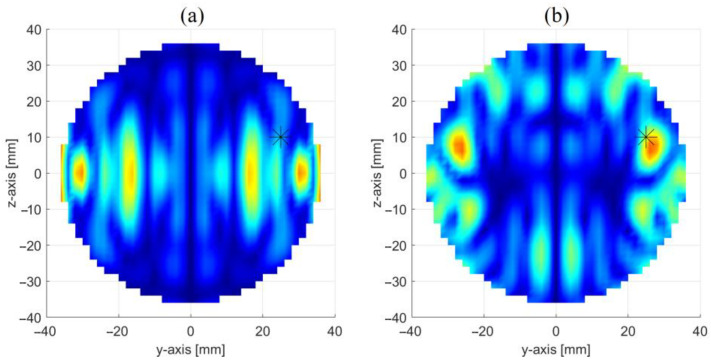
Reconstruction of the inclusion when the OUT is displaced of +5 mm along the *y*-axis: (**a**) data not calibrated; (**b**) data calibrated according to (5). (Stars give the position of the inclusion).

Regarding the PSP-based approach, Figure 10 shows |S_11_ − S_22_| (in dB) vs. frequency simulated in presence of the glass inclusion in the OUT. As it can be seen, the difference is much larger than the difference observed in the case of the perfectly centered OUT (see Figure 5), thus indicating that such a difference is mainly due to the displacement rather than the presence of the inclusion. This means that it is sufficient, a small displacement of the OUT from the center, to determine a completely loss of symmetry and so the failure of the PSP-based approach. This is confirmed by the map in Figure 11a representing the reconstructions obtained by processing the data in Figure 10. As it can be seen, the inclusion is completely undistinguishable, thus indicating that the effect of the displacement completely masks its presence.

However, the effect of possible displacement of the OUT from the center of the conveyor belt can be compensated by properly “calibrating” the measured S_11_ and S_22_. Denoted with S_11,0_ and S_22,0_, the reflection coefficients simulated for an OUT free of inclusions and perfectly centered between the two antennas, and with S_11,*d*_ and S_22,*d*_ the reflection coefficients simulated for the same OUT displaced of *d* along the *y*-axis, the calibration consists in rescaling the measured S_11_ and S_22_ as follow:

(5) $${\hat{S}}_{ii}=S_{ii}S_{ii,0}/S_{ii,d}\;\left(i=1,\;2\right)$$

The imaging is then performed by processing the calibrated differential data ${\hat{S}}_{11}-{\hat{S}}_{22}$ rather than to S_11_ − S_22_ (the data are still corrupted by an additive white Gaussian noise with a level of −65 dB).

Figure 11b shows the achieved reconstruction by means of the PSP-normalized approach. As it can be seen, apart from the ghost, the presence of the inclusion is again well distinguishable. Of course, the challenging aspect of such a calibration procedure is to know the exact displacement, *d*, of the OUT with respect to the antennas (this can be achieved, for instance, by exploiting the THz scanner/module proposed in [18]). Alternatively, in the absence of such information, one can determine S_11,*d*_ and S_22,*d*_ for a proper set of values of *d* and choose the couple (S_11,*d*_, S_22,*d*_), to be used in the calibration procedure in (5), such to minimize the root mean square error of ${\hat{S}}_{11}-{\hat{S}}_{22}$.

Another challenging aspect is to have a quite accurate numerical modeling of the OUT and of the system in order to accurately simulate *S_ii_*_,0_ and *S_ii_*_,*d*_ (*i* = 1, 2). In absence of this, one could physically measure (at the moment the system is deployed) *S_ii_*_,0_ and *S_ii_*_,*d*_ (*i* = 1, 2) for a reference object, for different *d*, and store the data for the subsequent calibration.

**Figure 12:**
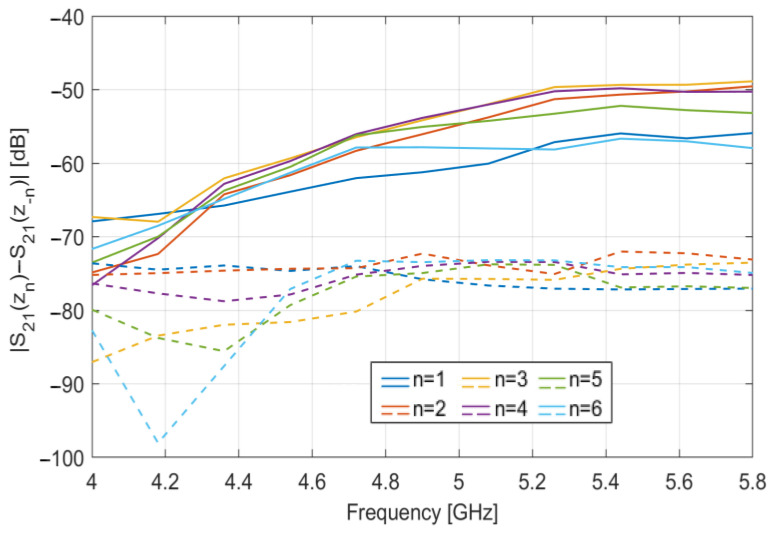
|S_21_(z*_n_*) − S_21_(z_−*n*_)| (in dB) vs. frequency simulated when the OUT is displaced of +5 mm along the *y*-axis: OUT with an 8 mm glass inclusion (solid lines); OUT free of inclusions (dashed lines).

As for the OSP-based approach, Figure 12 shows |S_21_(z*_n_*) − S_21_(z_−*n*_)| (in dB) vs. frequency simulated in presence (solid lines) and in absence (dashed lines) of the glass inclusion in the OUT. As it can be seen, in this case, a remarkable difference is still observed only when the inclusion is present, while it is practically zero in absence of it. Same behavior is observed for |S_11_(z*_n_*) − S_11_(z_−*n*_)| and |S_22_(z*_n_*) − S_22_(z_−*n*_)| but not reported for the sake of brevity. This confirms that, unlike the PSP-based approach, the detection capability of the OSP-based approach is not impaired by not perfectly centering OUT with respect to the two antennas. Therefore, at least when only the detection of the inclusion is of interest, the OSP-based approach is more robust than the PSP-based approach, as it does not require knowing possible displacements of the OUT from the middle between the two antennas. Therefore, it can be employed as a stand-alone approach of detection. Anyway, such information is still needed for the imaging, even if in this case we do not need to calibrate the data through (5), but the displacement must be taken into account solely in the computation of the Green’s matrixes in (4).

Finally, let us note that a similar analysis should be carried out by assuming not perfectly symmetric antennas. However, the effect of such asymmetry on the performances of the two approaches is analogous to that produced by a not perfect centering of the OUT between the two antennas, so that the above outcomes can be directly applied to this case.

In addition, let us note that a possible asymmetry in the behavior of the two antennas mainly arises from a not perfect symmetry of the elements connecting the antennas to the measurement instrument, such as adapters (e.g., microstrip to coaxial), tin welds and so on. Indeed, while the geometry/layout is almost the same for both antennas (thanks to the high mechanical tolerances of manufacturing, of the order of few tens of micron), the connection components, especially the tin welds, can be significantly different. However, in this study, not printed antennas (hence, without tin welds) were considered, which allows for minimizing this source of asymmetry. In any case, it is worth mentioning that possible residual asymmetries in the antennas’ behavior can be characterized and compensated or treated as a further source of noise, with the effect of increasing the level of the threshold above which the level of the differential data must be located to establish the presence of the inclusion. For instance, by indicatively assuming an (average) antenna return loss of approximately −25 dB, over the analyzed band (see Figure 4), and a discrepancy in the antenna behavior of approximately 3%, this corresponds to a difference between S_11_ and S_22_ without inclusion, of approximately −55 dB, which is larger than the considered noise level (−65 dB) but comparable to the level of the signal scattered by the considered inclusion (see Figure 5).

As final remark, let us note that, as happens in the case of a not perfect centering of the OUT between the two antennas, asymmetries in the antenna behavior only affects the performance of the PSP-based approach but not that of the OSP-based approach.

## 4. Conclusions

In this paper a simple MWI detection/imaging strategy enabling the in-line inspection of the possible presence of foreign bodies in packaged food, has been proposed. The strategy relies on the symmetries usually characterizing food items (and the radiating system), which can be broken by the presence of inclusions, thus determining a differential signal, revealing their presence. A quite common case, analyzed in this work, is that of homogeneous food packaged in a circular non-metallic jar. For this case, two approaches have been proposed, differing for the exploited symmetry: the PSP-based approach, which processes the difference arising in the reflection coefficients, measured at the two antennas, when the symmetry with respect to the PSP (i.e., the plane containing the direction of movement and virtually splitting the OUT into two symmetric parts) is broken by the presence of an inclusion; and the OSP-based approach which processes the difference arising in all the scattering parameters, measured for each couple of positions of the OUT along the direction of movement, when the symmetry with respect to the OSP (i.e., the plane orthogonal to the direction of movement and virtually splitting each antenna into two symmetric parts) is broken by the presence of an inclusion.

A comprehensive numerical analysis has shown the capability of both the approaches in detecting the inclusion as long as it does not lie on one of the symmetry planes characterizing each approach. A good reconstruction of the inclusion has also been achieved, except for the presence of a second “spot” in the mirror position, due to symmetrization. In any case, both of these drawbacks can be mitigated by combining the re-constructions achieved with both the approaches.

The robustness against possible displacements of the OUT from the middle between the two antennas has also been investigated. The analysis showed that the PSP-based approach is very sensitive to this, as the considered symmetry is also lost in the absence of inclusions. However, the approach still works provided to know, and hence to compensate, the displacement. On the contrary, at least for what concern the detection, the OSP-based approach is poorly sensitive to such error as the considered symmetry is not broken by such displacements. However, its knowledge is still required to achieve a reliable imaging of the inclusion. Accordingly, the OSP-based approach is preferable to the PSP-based approach at least when the only detection of the inclusion is of interest.

As final remark, it is worth remarking that the proposed approach works as long as the food product is (electrically) homogeneous. However, this condition could be not verified by the possible formation of air bubbles in the food during its pouring in the jar, which may render the food not homogeneous. Fortunately, this is issue can be easily eliminated by slightly shaking the object. Also, due to the negative electric contrast that air bubbles would induce in the food (being any type of food a medium electrically denser than air), they could be recognized as such, for instance, by employing, in combination, an algorithm of machine learning, as the one in [17].

In addition, we would like to stress that, while the presented study is still at numerical level, there are works in the literature wherein an experimental assessment (although preliminary) of a similar MWI tool has already been provided [10]. Indeed, in [10], apart from the imaging strategy (still differential, as in the present study, but exploiting an uncontaminated object as reference, rather than possible symmetries of the OUT, as in the present case), the experimental set up is practically the same as the one set in the simulations of this paper (as stressed in Section 2.3). This allows for being confident about the actual effectiveness of the approach and the success of its practical implementation. At present, an experimental characterization is currently under development. The aim is also to test the influence of all those factors (e.g., the non-uniformity of the jar wall thickness, the seam on a jar; non-parallelism of the upper surface of the contents and the conveyor belt and so forth) that could impair the performance of the approach.

## Figures and Tables

**Figure A1:**
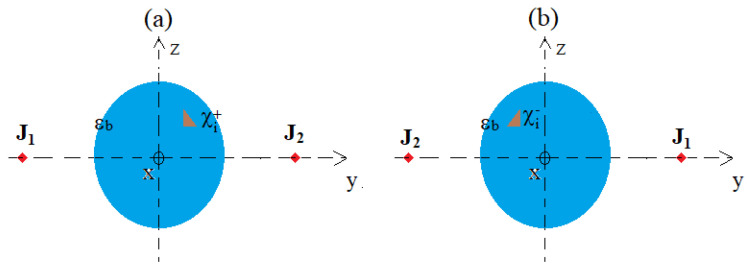
Conceptual scheme of the problem underlying the proposed differential MWI strategies: (**a**) point of view of the source J_1_; (**b**) point of view of the source J_2_.

## Data Availability

The data presented in this study are available on request from the corresponding author.

## Abbreviations

The following abbreviations are used in this manuscript.

MWI	MicroWave Imaging
OUT	Object Under Test
PSP	Parallel Symmetry Plane
OSP	Orthogonal Symmetry Plane
VNA	Vector Network Analyzer
TSVD	Truncated Singular Value Decomposition

## Appendix A

In this Appendix, we provide the theoretical background underlying the proposed differential MWI strategies presented in Section 2.2.1 and Section 2.2.2. For the sake of brevity, we only refer to the physical situation assumed for the PSP-based approach, as the situation assumed for the OSP-based approach can be dealt with in the same manner.

From an electromagnetic point of view, the situation at hand can be schematized as in Figure A1a: two identical current distributions, **J_1_** and **J_2_**, located at mirror position with respect to the *y*-axis (representing the two antennas in our system), radiating in an inhomogeneous background medium (BM), symmetric with respect to the *y*-axis (representing the OUT in our system), i.e., such that *ε_b_*(x, y, z) = *ε_b_*(x, −y, z), being *ε_b_* the relative electric permittivity. The symmetry of the BM is broken by the presence of an even small and weak electric inhomogeneity, *χ**_i_*^+^, located, for instance, in y > 0 (representing the inclusion).

Due to the symmetry, the point of view of **J_2_** is the same of that of **J_1_** except for *χ**_i_*^+^ which appears to **J_2_** at the mirror position of how it is appears to **J_1_** (see Figure A1b). Therefore, denoted with (**E_1_, H_1_**) the field radiated by **J_1_**, evaluated in a reference system with the *y*-axis pointing toward **J_2_** (see Figure A1a), and with (**E_2_, H_2_**) the field radiated by **J_2_**, evaluated in a reference system with the *y*-axis pointing toward **J_1_** (see Figure A1b), it is straightforward to show that the difference field **E** = **E_1_** − **E_2_**, **H** = **H_1_** − **H_2_** satisfies the following Maxwell’s equations (in the frequency domain):

(A1) $$\nabla \times E=-i\omega \mu_{0}H,$$

(A2) $$\nabla \times H=i\omega \varepsilon_{0}\varepsilon_{b}E+i\omega \varepsilon_{0}\varepsilon_{b}\chi_{i}^{+}E_{1}-i\omega \varepsilon_{0}\varepsilon_{b}\chi_{i}^{-}E_{2}$$

where *χ**_i_*^−^ is the mirror image of *χ**_i_*^+^ with respect to the *y*-axis. In (A2) **J_1_** and **J_2_** do not appear as, being equal and located in mirror position; they cancel each other.

In (A2) both **E_1_** and **E_2_** are in turn given by the superposition of the field radiated in the BM in absence of *χ**_i_*^+^ (i.e., for *χ**_i_*^+^ = 0), say **E_b_**, which is the same for both **J_1_** and **J_2_** (due to the symmetry of the BM), and by the field scattered by the *χ**_i_*^+^ and *χ**_i_*^−^, say **E_s1_** and **E_s2_**, respectively. Since *χ**_i_*^+^ is a small and weak scatterer, both **E_s1_** and **E_s2_** are negligible as compared to **E_b_**, so (A2) can be approximated as follows:

(A3) $$\nabla \times H=i\omega \varepsilon_{0}\varepsilon_{b}E+i\omega \varepsilon_{0}\varepsilon_{b}\chi_{i}^{+}E_{b}-i\omega \varepsilon_{0}\varepsilon_{b}\chi_{i}^{-}E_{b}$$

Since **E_b_** is assumed to be known, second and third terms on the right hand of (A3), say **J*_χi_*^+^** and **J*_χi_***^−^, can be regarded as the sources of the differential field (**E**, **H**), the support of which coincide with the support of *χ**_i_*^+^ and *χ**_i_*^−^. As a result, it is possible to reconstruct *χ**_i_*^+^ and *χ**_i_*^−^, hence the possible presence and the position of the inclusion, by solving a linear inverse problem starting from differential data, which is just what the proposed approach does.

In conclusion, we note that the presence of two current sources in (A2) implies that the solution of the inverse problem provides an image of both *χ**_i_*^+^ and its mirror counterpart *χ**_i_*^−^, which is exactly what we observe with the appearance of the “ghost” in the reconstructions of Section 3. In addition, when *χ**_i_* ^+^ = 0, i.e., in the absence of inclusion, (**E**, **H**) are solutions of a homogeneous Maxwell’s equation, therefore **E** = **0** and **H** = **0** in agreement with the fact that in the absence of inclusion, due to symmetry, the acquired differential data must be zero. Finally, (**E**, **H**) are the fields radiated by **J*_χi_*^+^** and **J*_χi_***^−^ in the BM *ε_b_* (i.e., in the presence of the OUT), so the reconstruction of *χ**_i_*^+^ and *χ**_i_*^−^ from field data, by solving the inverse problem, requires employing as Green’s function in the BM, i.e., **E_b_**, as executed in the proposed approach.
